# Radiobiological Meta-Analysis of the Response of Prostate Cancer to High-Dose-Rate Brachytherapy: Investigation of the Reduction in Control for Extreme Hypofractionation

**DOI:** 10.3390/cancers17081338

**Published:** 2025-04-16

**Authors:** Eva G. Kölmel, Miguel Pombar, Juan Pardo-Montero

**Affiliations:** 1 Group of Medical Physics and Biomathematics, Instituto de Investigación Sanitaria de Santiago (IDIS), 15706 Santiago de Compostela, Spain; 2 Department of Medical Physics, Complexo Hospitalario Universitario de Santiago de Compostela, 15706 Santiago de Compostela, Spain; 3 Department of Particle Physics, Universidade de Santiago de Compostela, 15705 Santiago de Compostela, Spain

**Keywords:** prostate cancer, HDR-BT, radiobiological modeling, dose–response models, LQ model, LQL model, reoxygenation

## Abstract

Background/Objectives: Clinical studies have shown a marked reduction in tumor control in prostate cancer treated with radically hypofractionated high-dose-rate brachytherapy (HDR-BT). The purpose of this study was to analyze the dose–response of prostate cancer treated with HDR-BT, specifically aiming at investigating the potential failure of the linear–quadratic (LQ) model to describe the response at large doses-per-fraction. Methods: We collated a dataset of dose–response to HDR-BT (3239 patients). The analysis was conducted separately for low and intermediate risk, resulting in 21 schedules (1633 patients) and 23 schedules (1606 patients), respectively. Data were fitted to tumor control probability models based on the LQ model, the linear–quadratic–linear (LQL), and a modification of the LQ model to include the effect of reoxygenation during treatment. Results: The LQ cannot fit the data unless the α/β is allowed to be high (∼[20, >100] Gy, 95% confidence interval). If the α/β is constrained to be low (≤8 Gy), the LQ model cannot reproduce the clinical results, and the LQL model, which includes a moderation of radiation damage with increasing dose, significantly improves the fitting. On the other hand, the reoxygenation model does not match the results obtained with the LQL. The clinically observed reduction in tumor control in prostate cancer treated with radical HDR-BT is better described by the LQL model. Using the best-fitting parameters, the BED for a 20 Gy × 1 treatment (128 ${\mathrm{Gy}}_{\alpha /\beta }$) is far less than that of a conventional 2 Gy × 37 fractionation (196 ${\mathrm{Gy}}_{\alpha /\beta }$). Conclusions: Our analysis showed that the substantial loss of tumor control observed in extremely hypofractionated HDR-BT trials can only be explained by the LQ model if the α/β is very large (≥100 Gy), in clear disagreement with the limits set in the analysis of external radiotherapy data. It seems more reasonable that there is a moderation of the LQ-predicted effect with increasing dose per fraction. These results may assist in the design of radical HDR-BT treatments.

## Simple Summary

Clinical studies have shown an important reduction in tumor control in prostate cancer when treated with radically hypofractionated high-dose-rate brachytherapy (HDR-BT). This poor response seems in contradiction with studies in external radiotherapy that showed that prostate cancer is very sensitive to fractionation (low α/β). The purpose of this study was to analyze the dose–response of prostate cancer treated with HDR-BT through biomathematical modelling, aiming at understanding the cause of the poor response for radically hypofractionated HDR-BT. We found that the LQ model cannot fit the dose–response curves and that models that include a moderation of the fractionation effect with increasing dose per fraction like the LQL provide a better fit to the experimental data. These results may assist in the design of radical HDR-BT treatments.

## 1. Introduction

Prostate cancer is one the most common cancers in men worldwide. Treatment options for localized prostate cancer include active surveillance, ablative radiotherapy, and radical prostatectomy [1]. Radiotherapy is widely used to treat prostate cancer. Radiotherapy options include external beam radiotherapy [2], proton therapy [3,4], and brachytherapy, either low-dose-rate or high-dose-rate (HDR-BT) [5,6]. No statistics are available on the use of each of these radiotherapy techniques to treat prostate cancer, and the relative use of each of them is likely hospital- and country-dependent. Nonetheless, HDR-BT is nowadays used as monotherapy for many patients [7], achieving good clinical outcomes, and because it is delivered with hypofractionated schedules, reducing treatment time and increasing patient comfort [6].

The response of prostate cancer to radiotherapy has been extensively studied [8,9,10]. The consensus is that the α/β ratio of prostate cancer is low (typically in the 1–4 Gy range), and therefore, this tumor is very sensitive to fractionation. Nonetheless, alternatives to the low α/β have been suggested, like tumor hypoxia [11]. In recent years, stereotactic body radiotherapy (SBRT) has become widely used to treat prostate cancer [12], with doses per fraction reaching up to 10 Gy. The response of prostate cancer to hypofractionated SBRT protocols has been recently analyzed [13,14,15]. All three studies reported low α/β ratios, in agreement with values obtained from lower doses per fraction. HDR-BT is delivered with hypofractionated protocols that are even more radical, reaching >20 Gy in a single fraction. However, several HDR-BT clinical trials have shown a marked reduction in tumor control (<70%) when delivering single-fraction treatments with >20 Gy. This loss in tumor control is not supported by a low α/β ratio. Guirado et al. have recently analyzed the response of prostate cancer to HDR-BT, suggesting a large α/β ratio (∼23 Gy) to explain the poor control achieved with HDR-BT single-fraction treatments [16]. They also argued that the linear–quadratic (LQ) model may not be adequate to describe the response to very large doses per fraction.

The validity of the LQ model for large doses per fraction has long been questioned [17], with different studies suggesting either a moderation or a boost of the cell-killing effect predicted by the LQ with increasing dose per fraction [18,19]. The moderation of the cell killing effect predicted by the LQ with increasing doses might explain the clinical results obtained with HDR-BT single-fraction treatments. This effect can be modeled with the linear–quadratic–linear (LQL) [20]. In fact, some evidence of an LQL-like response in the dose–response curves of prostate cancer treated with external radiotherapy was recently discussed in Refs. [14,15]. The poor control obtained with HDR-BT single-fraction treatments could also be rationally explained because of hypoxia and reoxygenation, as originally suggested by Nahum et al. [11]. If tumors are hypoxic and reoxygenate during treatment, short protocols delivering larger doses per fraction may be suboptimal.

In this work, we have collated and analyzed a dataset of dose–response for HDR-BT of prostate cancer. We have performed a radiobiological analysis of the dose–response, considering not only the LQ model but also more advanced models, including damage saturation at large doses and reoxygenation, aiming at advancing the understanding of the response of prostate cancer to very large doses per fraction.

## 2. Materials and Methods

### 2.1. Clinical Dataset

**Figure 1:**
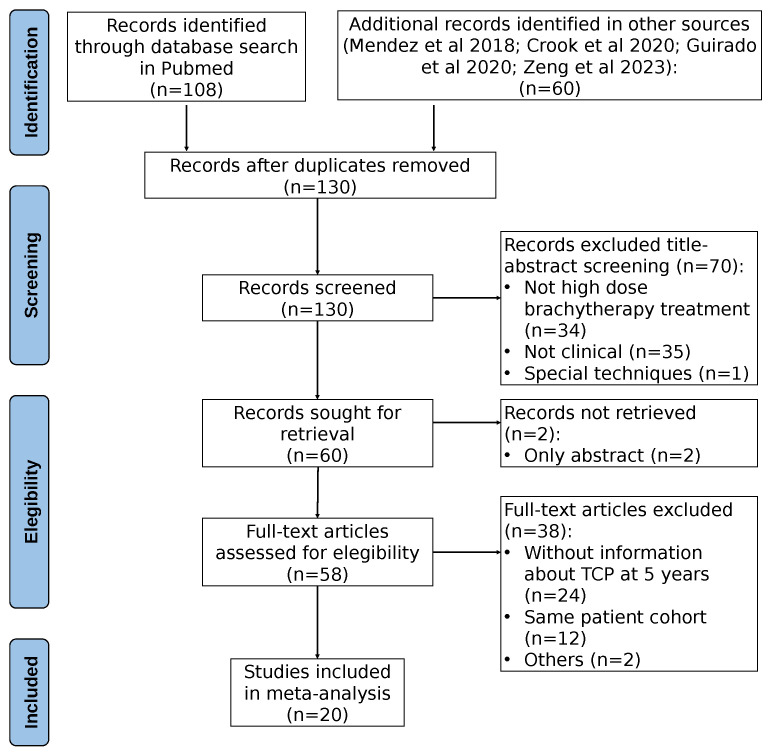
PRISMA chart of the bibliographic search.

The clinical dataset was created following a two-step process: initially, we expanded upon previously compiled datasets reported in [6,7,21]; subsequently, we performed a systematic search in Pubmed (in October 2024) for articles published after 2018 (the publication year of [6]). From each study, we extracted the number of patients, the distribution of patients according to the risk level, the number/percentage of patients receiving androgen deprivation therapy (ADT), the dose per fraction, the total dose, the overall treatment time and schedule details (fractions per day, time intervals between fractions), and the 5-year control rate, with control defined as freedom from clinical or biochemical failure (PSA nadir + 2 ng/mL). Studies that did not report any of these variables were excluded. Some studies included slightly different fractionations, and in those cases, the most commonly used fractionation was included. If different studies reported on the same (or similar) cohort but with different follow-up times, only the most recent publication was considered. The PRISMA chart of the bibliographic search is shown in Figure 1.

Overall, the collated dataset contained data from 20 studies (3239 patients) [22,23,24,25,26,27,28,29,30,31,32,33,34,35,36,37,38,39,40,41]. Our analysis was conducted separately for low risk (LR) and intermediate risk (IR), resulting in 21 schedules (1633 patients) for LR and 23 schedules (1606 patients) for IR. A limited number of data were found for high risk, but they were ignored because the number of schedules was not large enough to perform the analysis. Some studies stratified patients in more than three groups (e.g., “favorable intermediate risk”, “unfavorable intermediate risk” and “very low risk”). In such cases, these results were merged into a single group. The percentage of patients receiving ADT was included in the dataset, even though this variable was not used in this analysis.

**Figure 2:**
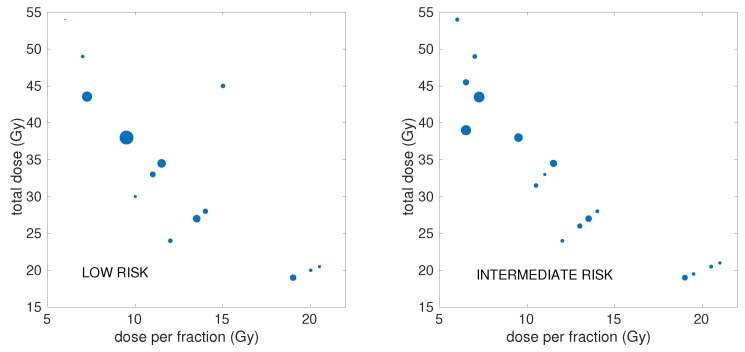
Overview of the characteristics of the schedules included in the analysis: total dose and dose per fraction for each of the different fractionations included in the dataset. Studies reporting on the same fractionation were merged for graphical display. Mark size is proportional to the number of patients.

An overview of the doses/doses-per-fraction and number of patients of the schedules included in the analysis is illustrated in Figure 2, with further detailed information presented in Supplementary Table S1.

### 2.2. Radiobiological Modeling

#### 2.2.1. Models

We used three different models to investigate the dose–response of prostate cancer to HDR-BT:

- The LQ model: The surviving fraction of tumor cells after a dose *d* is [42]
  (1) $$-\log SF_{LQ}=\alpha d+\beta d^{2}$$
- The LQL model: This model [20] includes a moderation of the quadratic term of the LQ model with increasing dose, which is controlled by the parameter δ:
  (2) $$-\log SF_{LQL}=\alpha d+\frac{2\beta }{\delta^{2}}\left(\delta d+\exp(-\delta d)-1\right)$$
- Stavrev’s model of reoxygenation: this model [43] relies on the LQ model, but with time-dependent α and β parameters to account for reoxygenation during treatment:
  (3) $$\alpha \left(t\right)=\alpha_{0}\exp\left(\frac{-bt^{2}}{2}\right)+\alpha_{0}\left(1+\phi \right)\left(1-\exp\left(\frac{-bt^{2}}{2}\right)\right)$$
  (4) $$\beta \left(t\right)=\beta_{0}{\left(\frac{\alpha (t)}{\alpha_{0}}\right)}^{2}$$
  with parameters *b* and ϕ controlling the evolution of α(t) and β(t). This results in a time-dependent α/β ratio, which, due to the quadratic dependence of β, increases with time.

#### 2.2.2. Incomplete Repair

Several schedules in the dataset delivered multiple fractions per day. In this situation, incomplete repair between consecutive fractions may play a role in the response to treatment. Therefore, we also investigated the possible contribution of incomplete repair by including it in the modeling. We used the LQ with incomplete repair correction [42,44]. The surviving fraction of cells following the *i*-th radiation fraction is given by

(5) $$\log SF_{i}=-\alpha d_{i}-\beta d_{i}^{2}-2\beta d_{i}\sum_{p=1}^{i-1}d_{p}\prod_{q=p}^{i-1}\theta_{p}$$

(6) $$\theta_{p}=\exp\left(-\nu \Delta t_{q}\right),\;\;\Delta t_{q}=t_{q+1}-t_{q}$$

where ν is the repair rate of sublethal damage (we will refer instead to the half-life of damage, defined as $T_{\mathrm{repair}}=\left(\log2\right)/\nu$).

Incomplete repair was also considered in the LQL and reoxygenation models, using an expression identical to Equation (5), but with $\alpha (t_{i})$ and $\beta (t_{i})$ replacing α and β for the reoxygenation model (where $t_{i}$ is the delivery time of the *i*-th fraction), and with an effective β-term replacing β for the LQL model, which can be obtained from Equation (2) as

(7) $$\beta_{\mathrm{eff}}=\frac{2\beta }{{\left(d\delta \right)}^{2}}\left(\delta d+\exp(-\delta d)-1\right)$$

#### 2.2.3. Overall Surviving Fraction and Proliferation

When delivering *n* fractions in an overall treatment time *T*, the surviving fraction is given by

(8) $$SF_{\mathrm{treatment}}=\left(\prod_{i=1}^{n}SF_{i}\right)\exp\left(\lambda \max\left(0,T-T_{k}\right)\right)$$

where $SF_{i}$ is the surviving fraction associated to each fraction, and proliferation is modeled as exponential with rate λ after a kick-off time $T_{k}$.

#### 2.2.4. Tumor Control Probability and EQD2

The tumor control probability (TCP) was modeled using a logistic function [45]:

(9) $$\mathrm{TCP}=\frac{1}{1+{\left(\frac{D_{50}}{\mathrm{EQD}2}\right)}^{4\gamma }}$$

where $D_{50}$ is the dose corresponding to 50% survival (in 2 Gy fractions), and γ controls the slope of the dose–response curve. The equivalent dose in 2 Gy fractions, EQD2, of each schedule is model-dependent, and its explicit calculation for the models used in this work is shown in the Supplementary Materials.

### 2.3. Statistical Methods

We fitted the models to the clinical data with the maximum likelihood methodology, assuming binomial statistics for the reported control. Different studies have different follow-up times, which can affect the statistical power of the study, as noted in [46]. In order to account for the follow-up, we converted the number of patients (*N*) in each schedule into an effective number of patients ($N_{\mathrm{eff}}$). The determination of $N_{\mathrm{eff}}$ is not exact, and has been performed by employing different quantitative and qualitative methods, including that presented in [46], which are reported in the Supplementary Materials. After correcting for follow-up, the total effective number of patients was $N_{\mathrm{eff}}$ = 1097 for LR (versus *N* = 1633) and $N_{\mathrm{eff}}$ = 1000 for IR (versus *N* = 1606). The value of $N_{\mathrm{eff}}$ (and *N*) for each schedule is reported in Supplementary Table S1.

We used an in-house-developed algorithm [15] based on the simulated annealing method to perform the optimization (minimization of −logL, where *L* is the likelihood). Confidence intervals (CIs) for the best-fitting parameters were obtained using the profile likelihood method [47].

Fits with the LQ model have five free parameters (α/β, $\lambda^{\prime }=\lambda /\alpha$, $T_{k}$, γ, $D_{50}$). For the LQL model, there is an extra free parameter, δ, and for Stavrev’s reoxygenation model, there are two extra parameters, ϕ and *b*. When including incomplete repair, there is an extra parameter for each model, $T_{\mathrm{repair}}$.

The space of parameter values was constrained to avoid reaching solutions that could be unphysical or not supported by biological data, and to speed up convergence. In particular, dose compensation due to accelerated proliferation was limited to $\lambda^{\prime }\leq$ 2 Gy ${\mathrm{day}}^{-1}$, a limit well higher than the proliferation found in [15], and the half-life of sublethal repair was limited to $T_{\mathrm{repair}}\leq$ 6 h. For the α/β ratio, we employed two different strategies due to the discrepancies on the reported values from external radiotherapy and HDR-BT: *Strategy 1*, a constraint 1 ≤α/β≤ 100 Gy to allow for large α/β ratios like those reported in [16]; and on the other hand, *Strategy 2*, a stronger constraint 1 ≤α/β≤ 8 Gy to force a low α/β ratio consistent with several reports from external radiotherapy.

The Akaike Information Criterion with sample size correction (${\mathrm{AIC}}_{c}$) was used to evaluate the performance of the models [48]:

(10) $$AIC_{c}=-2\log L+2k+\frac{2k(k+1)}{S-k-1}$$

where *k* is the number of parameters of the model, *S* is the sample size, and *L* is the maximum of the likelihood function. The difference in ${\mathrm{AIC}}_{c}$ of a given model compared to the reference model (the LQ model in this work), $\Delta {\mathrm{AIC}}_{c}^{\mathrm{model}}={\mathrm{AIC}}_{c}^{\mathrm{ref}}-{\mathrm{AIC}}_{c}^{\mathrm{model}}$, is an estimator of the relative quality of the model. Models with lower AICc are preferred, i.e., positive $\Delta {\mathrm{AIC}}_{c}$.

The results of model fitting to the experimental data were validated with a *Leave-One-Out* (LOO) cross-validation [49]. In this methodology, the models were repeatedly fitted to experimental sets $E_{i}$ containing S−1 points (*leaving one out*). This results in *S* different optimizations for each model and experimental set, which can be analyzed. We paid special attention to two different outputs: (i) the classification of the best model according to the Akaike Information Criterion for each optimization on $E_{i}$, aiming at determining whether model classification on the whole sample was particularly conditioned by the inclusion of one/some experimental point; and (ii) the dispersion of the best-fitting parameter values for each optimization on $E_{i}$ compared to the best-fitting parameter values on the whole experimental set.

We also investigated the parametric sensitivity of the models. We computed first- and total-order Sobol sensitivity indices following the methodology of Saltelli et al. [50]. More detailed information on the computation of the Sobol indices is presented in the Supplementary Materials.

The implementation of the methodology was performed in Matlab (Mathworks, Natick, MA, USA).

## 3. Results

**Figure 3:**
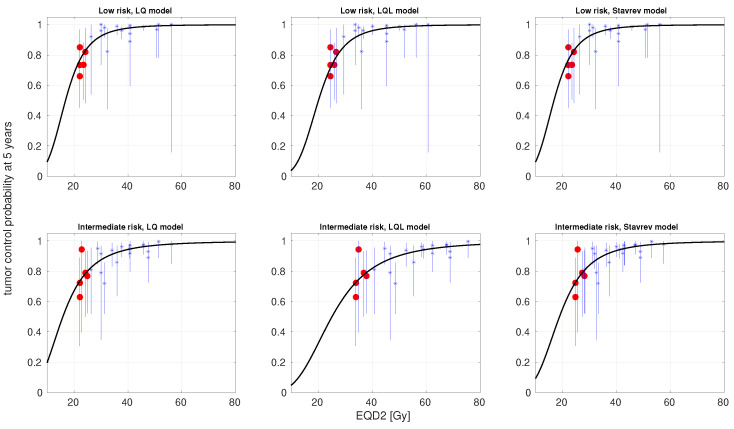
Best fits of LQ, LQL, and Stavrev’s reoxygenation models to dose–response data for prostate cancer treated with HDR-BT (low risk, top panels; intermediate risk, bottom panels). The α/β ratio was allowed to lie in a large interval (1 ≤α/β≤ 100 Gy). Clinical data (*); 95% confidence intervals (bars); and modeled curves (solid lines). Single-fraction schedules are highlighted as red circles.

In Table 1, we present the best-fitting parameters and the goodness-of-fit (−logL and ${\mathrm{AIC}}_{c}$) obtained from fitting the LQ, LQL, and Stavrev’s reoxygenation models (with and without incomplete repair correction) to low- and intermediate-risk data. For these fits, the α/β ratio was allowed to lie in a large interval (1 ≤α/β≤ 100 Gy). In Figure 3, we present the TCP-versus-EQD2 curves (experimental data and model best fits) obtained from this fitting strategy for the LQ, LQL, and reoxygenation models without incomplete repair correction. The 95% confidence intervals for the α/β and δ (LQL) are presented in Supplementary Table S2.

**Figure 4:**
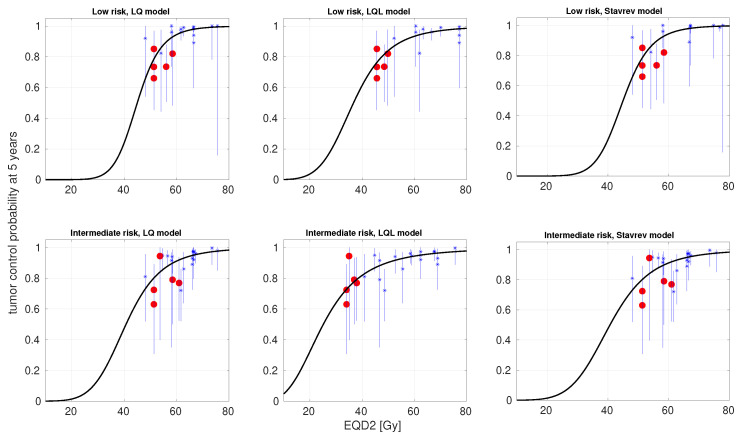
Best fits of LQ, LQL, and Stavrev’s reoxygenation models to dose–response data for prostate cancer treated with HDR-BT (low risk, top panels; intermediate risk, bottom panels). The α/β ratio was constrained to be low (1 ≤α/β≤ 8 Gy). Clinical data (*); 95% confidence intervals (bars); and modeled curves (solid lines). Single-fraction schedules are highlighted as red circles.

In Table 2, we present the best-fitting parameters and the goodness-of-fit for the same models fitted to the same data, but forcing the α/β ratio to be low (1 ≤α/β≤ 8 Gy), as many radiobiological studies in external radiotherapy support a low α/β ratio. In Table 3, we report the 95% confidence intervals of the best-fitting parameters for the latter scenario. The analysis of the confidence intervals, being computationally demanding, was limited to the LQ model with and without incomplete repair correction and the LQL model. In Figure 4, we present TCP-versus-EQD2 curves (experimental data and model best fits) for the LQ, LQL, and reoxygenation models without incomplete repair correction.

In Table 4, we present the results of the LOO cross-validation. This analysis was limited to the best-fitting models, the LQ model when the α/β ratio was allowed to lie in a large interval (Strategy 1), and the LQL model when the α/β ratio was constrained to be low (Strategy 2). In the table, we present the distribution of best-fitting parameters for each optimization (reported as mean and standard deviation), and the range of $\Delta {\mathrm{AIC}}_{c}^{\mathrm{LQL}}$ for each optimization (difference in ${\mathrm{AIC}}_{c}$ values between the LQ and LQL model).

First- and total-order Sobol sensitivity indexes are reported in Supplementary Table S3. The sensitivity analysis was also limited to the best-fitting models, the LQ model (Strategy 1), and the LQL model (Strategy 2).

## 4. Discussion

The response of prostate cancer to radiotherapy has been extensively studied, and the consensus is that the α/β ratio of prostate cancer is low [8,9,10,14,15]. This makes this tumor very sensitive to fractionation. Many external radiotherapy hypofractionated protocols have been investigated for prostate cancer [12]. Several studies have suggested that the LQ model may fail to describe tumor response at large doses per fraction [17,18,19] (even though the why and the how are not entirely clear, with different studies suggesting that the LQ may underestimate/overestimate the damage at large doses per fraction). In fact, recent analyses of the response of prostate cancer to SBRT reported a slight moderation of the LQ-predicted response at large doses [14,15].

The possible moderation of the damage with increasing dose is soft at the doses employed for SBRT, and clinical trials of SBRT for prostate cancer still reported high tumor control with doses per fraction up to 10 Gy [15]. This is not the case in HDR-BT, which is delivered with protocols that are more radical than those of SBRT, reaching >20 Gy in a single fraction. This makes HDR-BT an ideal scenario to investigate dose–response at large doses per fraction and the potential failure of the LQ model at such doses. Several HDR-BT clinical trials have shown a marked reduction in tumor control when delivering ∼20 Gy in a single fraction [22,23,24,25,26], including very recent studies [40,41]. This important loss in tumor control is not supported by a low α/β ratio and an LQ behavior at large doses: for example, assuming α/β = 3 Gy and using the LQ model, a 20 Gy × 1 treatment would be roughly isoeffective to 8 Gy × 5, and more effective than a conventional 2 Gy × 37 (BEDs of 153.3, 146.7, and 123.3 ${\mathrm{Gy}}_{3}$, respectively, ignoring proliferation). This led Guirado et al. to suggest a large α/β ratio in a recent analysis of prostate cancer response to HDR-BT [16]. However, such a large α/β ratio is not consistent with many radiobiological studies that found a low α/β ratio for prostate cancer. It may be that the LQ model is indeed not adequate to describe the response to very large doses per fraction [17].

Investigating the origin of the marked reduction in tumor control when delivering extremely hypofractionated HDR-BT seems of paramount importance to design effective treatments. In this study, we investigated the dose–response of prostate cancer to HDR-BT from a dataset containing 21 schedules (1633 patients) for LR and 23 schedules (1606 patients) for IR, with doses per fraction ranging from 6 to 21 Gy per fraction (Supplementary Table S1). Our analysis specifically focused on investigating the LQ and alternative models to characterize dose–response at such large doses. Because the clinical data point out an overestimation of the cell-killing effect by the LQ at large doses, we investigated the LQL model [20], which includes a moderation of cell killing at large doses. This particular response at large doses could also be caused by the role of hypoxia/reoxygenation, as originally suggested by Nahum et al. [11]. If reoxygenation plays an important role in the response to fractionated radiotherapy, extremely hypofractionated protocols might lose tumor control. To investigate the effect of reoxygenation, we have used the simple model proposed by Stavrev et al. [43], which accounts for reoxygenation through time-dependent α and β parameters. Because several schedules in the dataset delivered multiple fractions per day, we also investigated the role of incomplete repair on the modeling of response to treatment in each of the three models under investigation.

We followed two strategies for data fitting. First, we imposed broad constraints on the values of the best-fitting parameters. When following this strategy, the LQ proved superior to both the LQL and reoxygenation models to describe dose–response, with $\Delta {\mathrm{AIC}}_{c}^{\mathrm{LQL}}∼$−3 and $\Delta {\mathrm{AIC}}_{c}^{\mathrm{Sta}}∼$−8 (Table 1, Figure 3). However, in order to fit the data, the LQ led to a large α/β≥100 Gy (notice that this is the upper bound allowed in the optimization), with 95% confidence intervals α/β≥18 Gy and ≥17 Gy for LR and IR, respectively (Supplementary Table S2). This is an even larger value than that reported by Guirado et al. [16], who found α/β∼23 Gy from the analysis of a smaller dataset. The inclusion of incomplete repair in the modeling of response to treatment does not improve data fitting. For prostate cancer, studies have shown sublethal repair rates characterized by half-lives $T_{\mathrm{repair}}$∼1.5–2 h [51]. The effect of such a repair rate would be small for times between fractions ≥6 h, the typical time between fractions in the studies analyzed in this work.

The large α/β ratio obtained from this fitting strategy is not in agreement with many studies analyzing dose–response of prostate cancer treated with external radiotherapy. Therefore, we investigated a second fitting strategy where the value of the α/β ratio was constrained to be low, 1 ≤α/β≤ 8 Gy. These limits were qualitatively set to double the 95% confidence intervals reported in Ref. [15]. When forcing the α/β to be low, the results were quite different (Table 2, Figure 4), and the LQL model became superior to both the LQ and reoxygenation models, $\Delta {\mathrm{AIC}}_{c}^{\mathrm{LQL}}∼$8 for LR and 15 for IR. Analyses based on the AIC typically demand $\Delta {\mathrm{AIC}}_{c}$ > 10 to state the superiority of a given model [52]. In this case, the best-fitting α/β obtained with the LQL model is low (1 Gy; notice that this is the lower bound allowed in the optimization), but the whole search window is within the 95% CI. While the superiority of the LQL model over the LQ model is clear in this case, the analysis cannot properly separate the values of α/β and δ as seen in the corresponding 95% CI: a very low α/β and moderately low δ cannot be distinguished from a moderately low α/β and high δ. Using the best-fitting parameters of the LQL for LR (α/β = 1 Gy, δ = 0.31 ${\mathrm{Gy}}^{-1}$), the BED calculated with the LQL model for a 20 Gy × 1 treatment (128 ${\mathrm{Gy}}_{1}$) is far less than that of a conventional 2 Gy × 37 fractionation (196 ${\mathrm{Gy}}_{1}$).

An LOO cross-validation showed that these results are consistent. In particular, when following Strategy 1 (broad constraint on the α/β value), the LQ model with a large α/β value is systematically superior to the LQL model (44/44 optimizations), and when following Strategy 2 (α/β forced to be low), the LQL model with a very low α/β is superior to the LQ model (44/44 optimizations). A Sobol sensitivity analysis of these two models showed that they are particularly sensitive to the following (in decreasing order of sensitivity, see Supplementary Table S3): $D_{50}$ (which is given in units of EQD2 and therefore depends on the α/β), $\gamma_{50}$, δ (for the LQL), and α/β.

Interestingly, while the superiority of the LQL over the LQ is clear, the reoxygenation model does not improve the performance of the LQ model. This cannot be used to conclude that reoxygenation does not play a role in the response to HDR-BT, and may simply be due to the simplicity of the model considered. In particular, the implementation of the time variation of α and β due to reoxygenation is not dose- or treatment-dependent. The study of other more complex models accounting for hypoxia and reoxygenation has not been addressed in this work. For example, the models proposed by Kuperman and Lubich [53] or Jeong et al. [54].

The possibility that the LQ model may not be appropriate to describe dose–response at large doses per fraction has long been suggested [17,18]. In the context of prostate cancer, Refs. [14,15] found some evidence of a moderation of the LQ-predicted effect with increasing doses when analyzing clinical data of patients treated with external radiotherapy. HDR-BT is delivered with more radical protocols than SBRT, >20 Gy in a single fraction; therefore, the moderation of the effect can be more significant. Such radical hypofractionations are currently under investigation in external radiotherapy, with Zilli et al. currently investigating a 19 Gy × 1 fractionation [55]. The results of this and future clinical trials will shed more light on the response of prostate cancer to extreme hypofractionation.

In this study, we focused on studying potential radiobiological reasons for the decline in tumor control in extremely hypofractionated HDR-BT. A potential dosimetric origin of such low control (target coverage, dose homogeneity) was not investigated. Recently, Kuperman and Lubich [56] modeled the effect of target dose heterogeneities on the BED, and found that dose heterogeneities reduce the BED compared to the homogeneous dose scenario, especially for hypofractionated schedules. This effect might also explain the origin of the observed loss of tumor control for extreme hypofractionation, and merits further investigation.

Our study presents some limitations. In particular, the limited number of studies and the heterogeneity of the dataset may increase the uncertainties and potential sources of bias of the analysis by including different studies that may use different margins, different dose constraints, different dose calculation algorithms, etc. In particular, while the superiority of the LQL model over the LQ model seems clear, the analysis was not powerful enough to obtain narrow 95% CI for α/β and δ. Another limitation was that we only analyzed a limited number of dose–response models, as discussed above.

## 5. Conclusions

Several HDR-BT clinical trials have shown a marked reduction in tumor control in prostate cancer when treated with extremely hypofractionated protocols. Understanding the origin of this effect is of paramount importance to design effective treatments. Our analysis showed that the substantial loss of tumor control observed in extremely hypofractionated HDR-BT trials can only be explained by the LQ model if the α/β is very large (≥100 Gy), in clear disagreement with the limits set in the analysis of external radiotherapy data. It seems more reasonable that there is a moderation of the LQ-predicted effect with increasing dose per fraction, and in fact, if the α/β is constrained to be low (≤8 Gy), we found that the LQ model cannot fit the dose–response curves and the LQL proves to be the superior model. This is in agreement with recent studies of prostate cancer treated with external radiotherapy that found evidence of a moderation of the LQ-predicted effect with increasing dose per fraction. This moderation of the effect with increasing dose per fraction might affect the dose and fractionation prescription for prostate cancer.

The origin of the loss of control of radical single-fraction HDR-BT treatments merits further investigation; while in this work, a reoxygenation model did not fit the data as well as the LQL model, more complex reoxygenation models might provide better fits to the clinical data. Also, target dose heterogeneity may lead to patterns like those observed experimentally (loss of effectiveness for extreme hypofractionations), and should be further explored.

## Figures and Tables

**Table 1:** Best fits obtained with the LQ, LQL, and Stavrev’s reoxygenation model, ${\mathrm{LQ}}_{ST}$, without or with (subscript SD) sublethal damage incomplete repair correction, separated by risk (low, LR; and intermediate risk, IR). The table shows best-fitting parameters, maximum likelihood, ${\mathrm{AIC}}_{c}$, and $\Delta {\mathrm{AIC}}_{c}$ (referred to the LQ model) values. The symbol * indicates that the best-fitting parameter reached the edge of the constraint window.

Risk	Model	Parameters									Statistics		
		*α*/*β* [Gy]	*λ*′ [Gy day^−1^]	$T_{k}$ [day]	*δ* [Gy^−1^]	*ϕ*	*b* [h^−2^]	$T_{\mathrm{repair}}$ [h]	$D_{50}$ [Gy]	*γ*_50_	−*log*(*L*)	${\mathrm{AIC}}_{c}$	Δ ${\mathrm{AIC}}_{c}$
LR	LQ	100 *	0 *	-	-	-	-	-	16.83	1.09	36.71	87.41	-
	LQL	17.6	0.63	32.49	0.2	-	-	-	20.13	1.16	35.98	89.97	−2.56
	${\mathrm{LQ}}_{ST}$	100 *	0 *	-	-	0 *	0 *	-	16.83	1.09	36.71	96.03	−8.62
	${\mathrm{LQ}}_{SD}$	97.6	0.24	29.18	-	-	-	0 *	17.02	1.09	36.46	90.91	−3.5
	${\mathrm{LQL}}_{SD}$	4.6	1.17	35.06	0.24	-	-	0.05	26.89	1.28	35.80	94.22	−6.81
	${\mathrm{LQ}}_{ST,SD}$	93.0	1.03	37.64	-	0.12	$1.34\times 10^{-7}$	0.05	17.44	1.11	36.37	100.73	−13.32
IR	LQ	100 *	0 *	-	-	-	-	-	15.85	0.76	43.44	100.40	-
	LQL	1 *	0 *	-	0.53	-	-	-	25.12	0.81	43.08	103.41	−3.01
	${\mathrm{LQ}}_{ST}$	53.9	0.11	0.15	-	0.58	$7.73\times 10^{-7}$	-	18.87	0.90	43.33	108.12	−7.72
	${\mathrm{LQ}}_{SD}$	100 *	0 *	-	-	-	-	0 *	15.84	0.76	43.44	104.12	−3.72
	${\mathrm{LQL}}_{SD}$	1.3	0 *	-	0.54	-	-	0 *	23.69	0.80	43.09	107.64	−7.24
	${\mathrm{LQ}}_{ST,SD}$	100 *	0 *	-	-	0 *	0 *	0 *	15.85	0.76	43.44	113.16	−12.76

**Table 2:** Best fits obtained with the LQ, LQL, and Stavrev’s reoxygenation model, ${\mathrm{LQ}}_{ST}$, without or with (subscript SD) sublethal damage incomplete repair correction, separated by risk (low, LR; and intermediate risk, IR). The values of α/β were constrained to 1 ≤α/β≤ 8 Gy, to take into account the low α/β values typically reported for prostate cancer. The table shows best-fitting parameters, maximum likelihood, ${\mathrm{AIC}}_{c}$, and $\Delta {\mathrm{AIC}}_{c}$ (referred to the LQ model) values. The symbol * indicates that the best-fitting parameter reached the edge of the constraint window.

Risk	Model	Parameters									Statistics		
		*α*/*β* [Gy]	*λ*′ [Gy day^−1^]	$T_{k}$ [day]	*δ* [Gy^−1^]	*ϕ*	*b* [h^−2^]	$T_{\mathrm{repair}}$ [h]	$D_{50}$ [Gy]	*γ*_50_	−*log*(*L*)	${\mathrm{AIC}}_{c}$	Δ ${\mathrm{AIC}}_{c}$
LR	LQ	8 *	1	35.02	-	-	-	-	44.96	2.45	41.65	97.30	-
	LQL	1 *	1.33	27.00	0.31	-	-	-	36.71	1.34	35.63	89.25	8.05
	${\mathrm{LQ}}_{ST}$	8 *	1	35.02	-	0 *	0 *	-	44.96	2.45	41.65	105.91	−8.61
	${\mathrm{LQ}}_{SD}$	8 *	0.27	28.18	-	-	-	1.59	46.00	2.42	41.08	100.16	−2.86
	${\mathrm{LQL}}_{SD}$	1.9	0.44	29.11	0.38	-	-	0.26	28.93	1.27	36.03	94.68	2.62
	${\mathrm{LQ}}_{ST,SD}$	8 *	0.50	29.91	-	0.05	$4.02\times 10^{-7}$	0.05	44.61	2.35	41.67	111.34	−14.04
IR	LQ	8 *	0 *	-	-	-	-	-	40.66	1.50	52.21	117.95	-
	LQL	1 *	0 *	-	0.53	-	-	-	25.15	0.81	43.08	103.41	14.54
	${\mathrm{LQ}}_{ST}$	8 *	0 *	0.15	-	0 *	0 *	-	40.67	1.50	52.21	125.89	−7.94
	${\mathrm{LQ}}_{SD}$	8 *	0 *	-	-	-	-	4.00	41.59	1.27	45.86	108.96	8.99
	${\mathrm{LQL}}_{SD}$	1.28	1.51	47.65	0.45	-	-	1.47	26.65	0.82	42.89	107.25	10.7
	${\mathrm{LQ}}_{ST,SD}$	5.25	0.09	44.12	-	0.11	$1.94\times 10^{-6}$	4.23	44.00	1.34	45.52	117.32	0.63

**Table 3:** The 95% confidence intervals of best-fitting parameters for the LQ and LQL models without incomplete repair correction, and LQ model with incomplete repair correction (${\mathrm{LQ}}_{SD}$). Results are separated by risk—low (LR) and intermediate (IR). The symbol * indicates that the parameter value reached the edge of the constraint window.

Risk	Model	Parameters						
		*α*/*β* [Gy]	*λ*′ [Gy day^−1^]	$T_{k}$ [day]	*δ* [Gy^−1^]	$T_{\mathrm{repair}}$ [h]	$D_{50}$ [Gy]	*γ*_50_
LR	LQ	[6.7, 8 *]	[0 *, 2 *]	[0, 42 *]	-	-	[40.1, 49.5]	[1.76, 3.12]
	LQL	[1 *, 8 *]	[0 *, 2 *]	[0, 42 *]	[0.07, 1 *]	-	[12.6, 59.6]	[0.77, 2.14]
	${\mathrm{LQ}}_{SD}$	[6.2, 8 *]	[0 *, 2 *]	[0, 42 *]	-	[0 *, 3.69]	[38.9, 50.8]	[1.43, 3.04]
IR	LQ	[7.2, 8 *]	[0 *, 2 *]	[0, 42 *]	-	-	[32.8, 45.1]	[0.97, 2.04]
	LQL	[1 *, 8 *]	[0 *, 2 *]	[0, 42 *]	[0.06, 1 *]	-	[10.4, 49.4]	[0.50, 1.40]
	${\mathrm{LQ}}_{SD}$	[5.8, 8 *]	[0 *, 2 *]	[0, 42 *]	-	[2.03, 6 *]	[31.6, 51.1]	[0.72, 1.91]

**Table 4:** Results for the *Leave-One-Out* (LOO) cross-validation: We present mean and standard deviations of the best-fitting parameters for each optimization with S−1 points for the LR and IR groups. The results presented are limited to the best-fitting models, the LQ model for Strategy 1 (broad constraint in the value of α/β), and the LQL for Strategy 2 (α/β constrained to be low). We also report the values (range) of $\Delta {\mathrm{AIC}}_{\mathrm{LQL}}$ for the comparison of the LQL and LQ model, which show that the LQ is systematically better than the LQL for Strategy 1 and vice versa for Strategy 2.

	Strategy 1 (1 Gy ≤ α/β ≤ 100 Gy)		Strategy 2 (1 Gy ≤ α/β ≤ 8 Gy)	
	**LR - LQ**	**IR - LQ**	**LR - LQL**	**IR - LQL**
α/β [Gy]	90.11 ± 16.70	97.29 ± 11.95	2.22 ± 1.10	1.17 ± 0.80
$\lambda^{\prime }$ [Gy ${\mathrm{day}}^{-1}$]	0.20 ± 0.36	0.01 ± 0.04	0.93 ± 0.55	0.01 ± 0.05
$T_{k}$ [day]	13.29 ± 15.33	0.00 ± 0.00	26.69 ± 8.33	0.01 ± 0.05
$D_{50}$ [Gy]	17.43 ± 1.52	16.06 ± 1.01	30.49 ± 6.06	24.93 ± 3.41
$\gamma_{50}$	1.12 ± 0.01	0.78 ± 0.05	1.29 ± 0.15	0.81 ± 0.05
δ [${\mathrm{Gy}}^{-1}$]	-	-	0.36 ± 0.16	0.56 ± 0.13
$\Delta {\mathrm{AIC}}_{\mathrm{LQL}}$ [range]	[−3.53, −2.51]	[−3.58, −1.52]	[4.99, 11.12]	[7.91, 17.31]

## Data Availability

All data used in this work are available in the article and the Supplementary Materials.

## Supplementary Materials

The following supporting information can be downloaded at https://www.mdpi.com/article/10.3390/cancers17081338/s1: Supplementary Table S1: Detailed information of the analyzed schedules for low (LR) and intermediate risk (IR) prostate cancer, including: number of patients (N); effective number of patients ($N_{\mathrm{eff}}$) dose per fraction (*d*); number of fractions (*n*); total dose (*D*); irradiation schedule derived from the publications, and presented as the time in hours at which each fraction is delivered (for modeling incomplete repair between fractions); overall treatment time (*OTT*, defined as treatment time −1 day for modeling proliferation); percentage of patients receiving ADT, control at five years (*TCP*); and the first author and year of the study. Supplementary Table S2: 95% confidence intervals of best fitting parameters (α/β, δ) for the LQ and LQL models without incomplete repair correction. Results are separated by risk, low (LR) and intermediate (IR). The values of α/β were not constrained to be low (1 ≤α/β≤ 100 Gy). The symbol * indicates that the parameter value reached the edge of the constraint window. Supplementary Table S3: First-order, *S*, and total-order Sobol sensitivity indexes, $S_{T}$, for the fit of low-risk (LR) and intermediate-risk (IR) with the LQ model without incomplete repair correction when employing Strategy 1 (broad constraint on the value of α/β), and the LQL model without incomplete repair correction when employing Strategy 2 (α/β constrained to be low). Supplementary Methodology: calculation of the EQD2 for different models; follow-up and effective number of patients; sensitivity analysis.
