# Supplementary material for: Radiobiological Meta-Analysis of the Response of Prostate Cancer to High-Dose-Rate Brachytherapy: Investigation of the Reduction in Control for Extreme Hypofractionation
Source: Cancers (Basel). 2025 Apr 16;17(8):1338. doi: 10.3390/cancers17081338 (PMC12025418; doi:10.3390/cancers17081338)
Supplement: Supplementary file 1 [file cancers-17-01338-s001.zip › cancers-3538639-supplementary.pdf]

*Supplementary Materials for "**Radiobiological meta-analysis of the response of prostate cancer to high dose rate brachytherapy**"*

1. Supplementary methodology

1.1. Calculation of the EQD2 for different models

The *equivalent dose in 2 Gy fractions, EQD2,* of a given schedule is calculated by imposing iso-effectiveness with a 2 Gy/fraction treatment. There is some ambiguity in the calculation of the EQD2 regarding the effects that are included in the computation of the effectiveness of the 2 Gy/fraction treatment. Here, we will follow **[14]** and include *dose effects* (like the moderation of the quadratic term with increasing dose in the LQL model), but will ignore *time effects* (like incomplete repair or proliferation), which would require to assign a given schedule to the 2 Gy/fraction treatment (e.g. weekends off or not).

Below we present the analytical form of the EQD2 for each model. In these equations, d is the dose per fraction, n is the number of fractions, and D is the total dose:

LQ model

$$EQD2_{LQ}=\frac{\left(D+\frac{dD}{(\alpha/\beta)}-\left(\frac{\lambda}{\alpha}\right)max\left(0,T-T_k\right)\right)}{\left(1+\frac{2}{(\alpha/\beta)}\right)} \quad \text{(SM1)}$$

If we include the effect of sublethal damage incomplete repair we obtain:

$$EQD2_{LQ,SD}=\frac{\left(D+\frac{dD}{(\alpha/\beta)}+\frac{2d^2\sum_{i=1}^{n}\left(\sum_{p=1}^{i-1}\left(\prod_{q=p}^{i-1}\theta_q\right)\right)}{(\alpha/\beta)}-\left(\frac{\lambda}{\alpha}\right)\max\left(0,T-T_k\right)\right)}{\left(1+\frac{2}{(\alpha/\beta)}\right)} \quad \text{(SM2)}$$

LQL model

$$EQD2_{LQL}=\frac{\left(D+\frac{2\left(\delta d+\exp\left(-\delta d\right)-1\right)D}{\left(\alpha/\beta\right)d\delta^2}-\left(\frac{\lambda}{\alpha}\right)max\left(0,T-T_k\right)\right)}{\left(1+\frac{2\delta+\exp\left(-2\delta\right)-1}{\left(\alpha/\beta\right)\delta^2}\right)} \quad \text{(SM3)}$$

If we include the effect of sublethal damage incomplete repair we obtain:

$$EQD2_{LQL,SD}=\frac{\left(D+\frac{dD\left(\delta d+\exp\left(-\delta d\right)-1\right)}{\left(\alpha/\beta\right)\left(\delta d\right)^2}+\frac{2d^2\left(\delta d+\exp\left(-\delta d\right)-1\right)\sum_{i=1}^{n}\left(\sum_{p=1}^{i-1}\left(\prod_{q=p}^{i-1}\theta_q\right)\right)}{\left(\alpha/\beta\right)\left(\delta d\right)^2}-\left(\frac{\lambda}{\alpha}\right)\max\left(0,T-T_k\right)\right)}{\left(1+\frac{\left(2\delta+\exp\left(-2\delta\right)-1\right)}{\left(\alpha/\beta\right)\delta^2}\right)} \quad \text{(SM4)}$$

Re-oxygenation model

$$EQD2_S=\frac{\frac{1}{\alpha_0}\left(\sum_{i=1}^{n}\alpha_i d_i+\sum_{i=1}^{n}\beta_i d_i^2-\lambda max\left(0,T-T_k\right)\right)}{1+\frac{2}{\left(\alpha_0/\beta_0\right)}} \quad \text{(SM5)}$$

where α_i and β_i refer to the values of α and β at the time $t=t_i$ of delivery of the i-th fraction.

If we include the effect of sublethal damage incomplete repair we obtain:

$$EQD2_{S,SD}=\frac{\frac{1}{\alpha_0}\left(\sum_{i=1}^{n}\alpha_i d_i+\sum_{i=1}^{n}\beta_i d_i^2+2\sum_{i=1}^{n}\beta_i d_i\left(\sum_{p=1}^{i-1}d_p\left(\prod_{q=p}^{i-1}\theta_q\right)\right)-\lambda max\left(0,T-T_k\right)\right)}{1+\frac{2}{\left(\alpha_0/\beta_0\right)}} \quad \text{(SM6)}$$

1.2. Follow-up and effective number of patients

In this work we have analyzed the tumor control probability (TCP) at five years with the maximum likelihood methodology, assuming binomial statistics for the reported control according to the number of patients reported for each cohort (N). However, in the clinical studies not all patients included in each cohort reach the follow up time of five years due to censoring. Therefore, a statistical meta-analysis considering N patients for a given cohort may overestimate the contribution of that cohort if the case of a short follow-up.

Huang et al. noticed this in Ref. [46] and presented an analytical method to obtain the effective number of patients, N_{eff}, for a cohort as:

$$N_{eff} = TCP(1-TCP)\left(\frac{3.92}{CI}\right)^2 \quad \text{(SM7)}$$

where *TCP i*s the reported tumor control probability and CI is the 95% confidence interval.

This expression provides a simple closed-form method to compute N_{eff} for each cohort. However, this expression combines elements from the binomial distribution, the normal distribution, and the Kaplan-Meier estimator (the CI reported in the clinical studies usually comes from the Kaplan-Meier estimator and the Greenwood formula to calculate the variance of the estimator). Because of this, the application of the above formula to compute N_{eff} in somes cases yields N_{eff} >N, especially in situations where the number of patients is low, where the CIs obtained from the binomial distribution are narrower than those obtained from the Greenwood formula.

In this study, we have followed a mixed quantitative/qualitative approach to obtain the value of N_{eff} for each cohort, based on the analysis of reported follow-ups, Kaplan-Meier curves, and Huang's method (Equation SM7). Four different procedures were used, which are reported below in order of preference:

Case 1 - Quantitative analysis of follow-up statistics and/or KM curves: If the minimum follow up time, FU_{min}, is reported in the article and FU_{min} ≥ 5y, then N_{eff}=N. If the study reports the number/fraction of patients with FU≥5y, the value of N_{eff} is computed accordingly. If the KM curve reports the number of censoring events before 5y, *c*, or it can be inferred from the reported data (i.e. patients at risk), we use N_{eff} = N-*c*.

Case 2- Huang's method: Otherwise, we used reported CI (or extract them from the KM curve with the IPDfromKM package) and applied Huang's method (Equation SM7) to obtain N_{eff}. If the value obtained for N_{eff} is not reliable (N_{eff} >N), we rely on qualitative estimations of N_{eff}.

Case 3 – Qualitative analysis of KM curves: We use a visual inspection of the KM curves to count the number of failure events (f). We then approximate N_{eff} as TCP≈(N_{eff} – f)/N_{eff}, N_{eff} ≈ f/(1-TCP). This qualitative method can be easily performed for trials involving a relatively low number of patients.

Case 4 - Qualitative analysis of follow-up statistics: In a few cases none of the above three methods could be used to obtain a value of N_{eff}. In such situation, we used the reported median of the follow-up distribution to compute $N_{eff.}$ A few studies reported median follow-up ≈ 5y, which lead us to consider N_{eff} = N/2.

In some cases, for studies reporting results for low- and intermediate-risk patients, the reported data only allowed to compute N_{eff} for the whole population. In such cases, the effective number of patients for each risk group was calculated by applying the ratio (N/N_{eff}) to each risk group, $N_{eff,risk} = N_{risk} * N/N_{eff}$.

1.3. Sensitivity analysis: Sobol indices

We used the methodology of Sobol indices to investigate the behavior of the model under the perturbation of its parameters. The analysis was limited to the best-fitting models, i.e. the LQ model when employing Strategy 1 (broad constraint in the value of α/β), and the LQL for Strategy 2 (α/β constrained to be low). The first- and total- order sensitivity indexes (S_i and $S_{T,i}$) were calculated for each model parameter.

The first- and total- order sensitivity indices were computed following the methodology of Saltelli et al. Two matrices of N x K randomly perturbed parameters, A and B, were computed (the model parameters were perturbed following a normal distribution with relative standard deviation of 0.1). K is the number of parameters and N is the number of experiments, which was set to 10^5. Additional matrices were defined as $C_i = A$, except for the i-th column which equals the i-th column of B. From each parameter combination present in these matrices, the output from the model was obtained in the form of N x 1 vectors:

$$y_A = f(A) \qquad y_B = f(B) \qquad y_{C_i} = f(C_i) \tag{SM8}$$

Taking into account this notation, the Sobol indices were calculated as:

$$V = \frac{1}{2N}\sum_{j=1}^{N}\left(y_{A,j}^2 + y_{B,j}^2\right) - \left(\frac{1}{2N}\sum_{j=1}^{N}\left(y_{A,j} + y_{B,j}\right)\right)^2 \tag{SM9}$$

$$S_i = \left[\frac{1}{N}\sum_{j=1}^{N}\left(y_{B,j}\left(y_{C_i,j} - y_{A,j}\right)\right)\right]/V \tag{SM10}$$

$$S_{T,i} = \left[\frac{1}{2N}\sum_{j=1}^{N}\left(y_{A,j} - y_{C_i,j}\right)^2\right]/V \tag{SM11}$$

Values of S and S_T are presented in Supplementary Table 3.

2. Supplementary Tables

Supplementary Table S1: Detailed information of the analyzed schedules for low (LR) and intermediate risk (IR) prostate cancer, including: number of patients (N); effective number of patients (N_{eff}) dose per fraction (d); number of fractions (n); total dose (D); irradiation schedule derived from the publications, and presented as the time in hours at which each fraction is delivered (for modeling incomplete repair between fractions); overall treatment time (OTT, defined as treatment time -1 day for modeling proliferation); percentage of patients receiving ADT, control at five years (TCP); and the first author and year of the study.

Risk	N	N_{eff}	d (Gy)	n	D (Gy)	Schedule (hours)	OTT (days)	ADT (%)	TCP (%)	Reference
LR	288	218[α]	7.25	6	43.5	[0 6 24 168 174 192]	8	0.0[a]	98.7	Hauswald (2016)
LR	19	10[β]	10.0	3	30.0	[0 6 24]	1	26.3	82.3	Barkati (2012)
LR	198	121[β]	11.5	3	34.5	[0 504 1008]	42	5.0	96.1	Strouthos (2018)
LR	47[g]	23[γ]	15.0	3	45.0	[0 480 984]	41[k]	87.0	96.7	Kukielka (2015)
LR	233	116[γ]	9.5	4	38.0	[0 6 24 30]	1	0.0[b]	98.0	Jawad (2016)
LR	48	6[β]	12.0	2	24.0	[0 6]	0[f]	0.0[c]	92.0	Jawad (2016)
LR	56	23[β]	13.5	2	27.0	[0 6]	0[f]	0.0[d]	100.0	Jawad (2016)
LR	44	31[α]	19.0	1	19.0	[0]	0	34.0	66.0	Prada (2016)
LR	103	103[δ]	9.5	4	38.0	[0 24 30 48]	2	0.0[e]	99.4	Behmueller (2021)
LR	15[f]	15[δ]	9.5	4	38.0	[0 6 24 30]	1	0.0	89.0	Johansson (2021)
LR	85	81[α]	11.0	3	33.0	[0 336 672]	27	0.0	99.0	Johansson (2021)
LR	69	63[α]	14.0	2	28.0	[0 336]	13	0.0	98.0	Johansson (2021)
LR	21	20[α]	19.0	1	19.0	[0]	0	0.0	85.1	Hudson (2024)
LR	12	12[α]	13.5	2	27.0	[0 168]	7	0.0	100.0	Hudson (2024)
LR	196	98[γ]	9.5	4	38.0	[0 6 336 342]	13	3.6	94.0	Tselis (2013)
LR	84[h]	84[δ]	13.5	2	27.0	[0 6]	0	32.7	96.0	Nagore (2023)
LR	26	15[α]	7.0	7	49.0	[0 6 24 30 48 54 72]	3	7.7	100.0	Yamazaki (2018)
LR	2	2[δ]	6.0	9	54.0	[0 6 24 30 48 54 72 78 96]	4	0.0	100.0	Yamazaki (2018)
LR	22	11[γ]	20.5	1	20.5	[0]	0	68.2	82.0	Prada (2018)
LR	25[i]	25[δ]	20.0	1	20.0	[0]	0	0.0	73.5	Hannoun-Levi (2022)
LR	40[j]	20[γ]	19.0	1	19.0	[0]	0	0.0	73.4	Siddiqui (2019)

[a] 42 patients out of 448 (288 LR and 160 IR) received ADT. Because ADT is most likely prescribed to HR/IR patients, we assumed that 0/288 LR patients received ADT.

[b] 61 patients out of 319 (233 LR, 86 IR) received ADT. Because ADT is most likely prescribed to HR/IR patients, we assumed that 0/233 LR patients received ADT.

[c] 4 patients out of 79 (48 LR, 31 IR) received ADT. Because ADT is most likely prescribed to HR/IR patients, we assumed that 0/48 LR patients received ADT.

[d] 3 patients out of 96 (56 LR, 40 IR) received ADT. Because ADT is most likely prescribed to HR/IR patients, we assumed that 0/96 LR patients received ADT.

[e] 33 patients out of 141 (103 LR, 32 IR, 6 HR) received ADT. Because ADT is most likely prescribed to HR/IR patients, we assumed that 0/103 LR patients received ADT.

[f] 15 out fo 19 were LR. Biochemical control (BC) was not specified for LR and IR, and we assigned these results to LR.

[g] 47 out of 77 patients were LR (61%). BC was not specified by risk group, and we assigned the overall BC (96.7%) to LR.

[h] 84 out of 119 patients were LR (71%). BC was not specified by risk group, and we assigned the overall BC (96.0%) to LR.

[i] 25 out of 33 were LR (76%). BC was not specified by risk group, and we assigned the overall BC (73.5%) to the LR group.

[j] 40 out of 68 patients were LR (59%). BC was not specified by risk group, but the study found “No significant difference between low- and intermediate-risk patients”. We assigned the overall BC (73.5%) to the LR group.

[k] Median value

[f] Two schedules were employed, two fractions in a day separated > 6 h or two fractions separated 2 weeks. We do not have enough data to differenciate between them, and we assigned the results to the former.

[α] Estimation from the KM plot.

[β] Huang's method

[γ] Neff = N/2 because FU ≈ 5 y

[δ] Neff = N because FU_{min} > 5 y (or median FU >> 5 y)

Risk	N	N_{eff}	d (Gy)	n	D (Gy)	Schedule (hours)	OTT (days)	ADT (%)	TCP (%)	Reference
IR	49[m]	33[α]	6.5	7	45.5	[0 6 24 30 48 54 72]	3	44.3	93.0	Yoshioka (2016)
IR	160	104[α]	7.25	6	43.5	[0 6 24 168 174 192]	8	26.3	97.6	Hauswald (2016)
IR	284	110[β]	6.5	6	39.0	[0 5 24 408 413 432]	18[p]	16.2	94.4	Rogers (2012)
IR	158[n]	97[ε]	7.25	6	43.5	[0 6 24 168 174 192]	8	0.0	97.0	Patel (2017)
IR	135	95[β]	11.5	3	34.5	[0 504 1008]	42	11.9	96.1	Strouthos (2018)
IR	86	43[γ]	9.5	4	38.0	[0 6 24 30]	1	70.9	95.0	Jawad (2016)
IR	31	15[γ]	12.0	2	24.0	[0 6]	0[q]	12.9	81.0	Jawad (2016)
IR	40	8[β]	13.5	2	27.0	[0 6]	0[q]	7.5	79.0	Jawad (2016)
IR	28	4[α]	19.5	1	19.5	[0]	0	53.6	94.4	Hoskin (2017)
IR	69	44[α]	13.0	2	26.0	[0 6]	0	52.2	95.0	Hoskin (2017)
IR	49	49[α]	10.5	3	31.5	[0 6 24]	1	71.0	94.0	Hoskin (2017)
IR	32	32[δ]	9.5	4	38.0	[0 24 30 48]	2	84.4	97.2	Behmueller (2021)
IR	22	21[α]	11.0	3	33.0	[0 336 672]	27	0.0	86.0	Johansson (2021)
IR	34	31[α]	14.0	2	28.0	[0 336]	13	0.0	72.0	Johansson (2021)
IR	66	60[α]	19.0	1	19.0	[0]	0	0.0	72.4[o]	Hudson (2024)
IR	71	68[α]	13.5	2	27.0	[0 168]	7	0.0	91.5[o]	Hudson (2024)
IR	81	40[γ]	9.5	4	38.0	[0 6 336 342]	13	23.5	92.0	Tselis (2013)
IR	48	34[α]	6.5	7	45.5	[0 6 24 30 48 54 72]	3	22.9	89.0	Yamakazi (2018)
IR	52	31[α]	7.0	7	49.0	[0 6 24 30 48 54 72]	3	96.2	99.6	Yamakazi (2018)
IR	39	35[α]	6.0	9	54.0	[0 6 24 30 48 54 72 78 96]	4	76.9	97.8	Yamakazi (2018)
IR	34	17[γ]	20.5	1	20.5	[0]	0	14.7	79.0	Prada (2018)
IR	16	11[α]	19.0	1	19.0	[0]	0	31.3	63.0	Prada (2016)
IR	22[r]	18[α]	21.0	1	21.0	[0]	0	0.0	76.9	Salari (2024)

[m] 62% of the 79 patients were treated with this treatment plan. Other fractionations were employed, but since BC is not specified separately, we assigned the overall BC (93%) to this fractionation.

[n] 83% of the patients received this treatment plan. Other fractionations were employed, but since BC is not specified separately, we assigned the overall BC (93%) to this fractionation.

[o] Weighted average of the BC for IR favourable group and IR unfavourable group.

[p] Mean value

[q] Two schedules followed: two sessions in a day separated > 6 h or two sessions separated 2 weeks. We don't have enough data to differenciate it, and we choose the first one

[r] 22 out of 26 were IR patients. BC was not specified by risk group, and we assigned the overall BC to the IR group.

[α] Estimation from the KM plot.

[β] Huang's method

[γ] Neff = N/2 because FU ≈ 5 y

[δ] Neff = N because FUmin > 5 y or median FU >> 5 y

[ε] 61% of the patients had FU > 5 y

Supplementary Table S2: 95% confidence intervals of best fitting parameters (*α/β*, *δ*) for the LQ and LQL models without incomplete repair correction. Results are separated by risk, low (LR) and intermediate (IR). The values of *α/β* were not constrained to be low (1 ≤ *α/β* ≤ 100 Gy). The symbol * indicates that the parameter value reached the edge of the constraint window.

**Risk**	**Model**	**Parameters**	
		***α/β* [Gy]**	***δ* [Gy^{-1}]**
**LR**	**LQ**	[18.5, 100*]	-
	**LQL**	[1*, 100*]	[0*, 1*]
**IR**	**LQ**	[16.9, 100*]	-
	**LQL**	[1*, 100*]	[0*, 1*]

Supplementary Table S3: First-order, *S*, and total-order Sobol sensitivity indexes, S_T, for the fit of low-risk (LR) and intermediate-risk (IR) with the LQ model without incomplete repair correction when employing Strategy 1 (broad constraint on the value of *α/β*), and the LQL model without incomplete repair correction when employing Strategy 2 (*α/β* constrained to be low).

	**LR - LQ**		**IR - LQ**		**LR - LQL**		**IR - LQL**	
	**S**	**S_T**	**S**	**S_T**	**S**	**S_T**	**S**	**S_T**
**α/β**	0.000	0.009	0.000	0.006	0.000	0.024	0.000	0.011
**λ’**	0.000	0.010	0.010	0.016	0.000	0.001	0.000	0.000
**T_k**	0.003	0.007	0.002	0.007	0.000	0.002	0.000	0.000
**D_{50}**	0.563	0.892	0.396	0.840	0.463	0.874	0.354	0.824
**$γ_{50}$**	0.106	0.445	0.137	0.554	0.033	0.236	0.071	0.453
δ	-	-	-	-	0.032	0.2911	0.009	0.210
